# The Endoscope

**Published:** 1868-02-15

**Authors:** 


### EDITORIAL.

BACK NOS. WANTED.—*Owing to the large increase in our subscription list, although it was supposed a sufficient number of copies had been provided, we are entirely out of copies of the* JOURNAL *for the months of* JANUARY, JUNE, SEPTEMBER, OCTOBER, *and* NOVEMBER, 1867, *Vol.* XXIV. *We will pay twenty-five cents each for copies of either of these, in cash or by credit on the present volume, as desired. Will our friends who do not file their numbers oblige us by responding?*

### *The Endoscope.*

Subscribers, or others, who have not as yet received their copies of the *Endoscope* as ordered, will receive them in a few days, the present supply being exhausted.

